# Identification of early coagulation changes associated with survival outcomes post severe burns from multiple perspectives

**DOI:** 10.1038/s41598-024-61194-0

**Published:** 2024-05-07

**Authors:** Shengyu Huang, Qimin Ma, Xincheng Liao, Xi Yin, Tuo Shen, Xiaobin Liu, Wenbin Tang, Yusong Wang, Lei Wang, Haiming Xin, Xiaoliang Li, Liu Chang, Zhaohong Chen, Rui Liu, Choulang Wu, Deyun Wang, Guanghua Guo, Feng Zhu

**Affiliations:** 1 Medical Center of Burn Plastic and Wound Repair, The First Affiliated Hospital, Jiangxi Medical College, Nanchang University, Nanchang, 330006 Jiangxi Province China; 2 Department of Critical Care Medicine, Shanghai East Hospital, School of Medicine, Tongji University, Shanghai, 200120 China; 3 Department of Burns and Plastic Surgery, Zhangjiagang First People’s Hospital, Suzhou, 215600 China; 4 Department of Burns, Guangzhou Red Cross Hospital, Jinan University, Guangzhou, 510220 China; 5 ICU of Burn and Trauma, the First Affiliated Hospital of Naval Medical University, Shanghai, 200433 China; 6 Department of Burns and Plastic Surgery, Affiliated Hospital of Nantong University, Nantong, 226001 China; 7 Department of Burns and Plastic Surgery, The 924th Hospital of the Chinese People’s Liberation Army, Guilin, 541002 China; 8 Department of Burns and Plastic Surgery, Zhengzhou First People’s Hospital, Zhengzhou, 450004 China; 9 Department of Burns and Plastic Surgery, the Fourth People’s Hospital of Dalian, Dalian, 116031 China; 10 Department of Burns, Fujian Medical University Union Hospital, Fuzhou, 350001 China; 11 Department of Burn Surgery, Heilongjiang Provincial Hospital, Harbin, 150036 China; 12 Department of Burns, Taizhou Hospital of Zhejiang Province, Linhai, 317000 China; 13 Department of Burns, Wuhan Third Hospital, Wuhan, 430060 China

**Keywords:** Trauma, Risk factors, Outcomes research

## Abstract

Coagulation alterations manifest early after severe burns and are closely linked to mortality outcomes. Nevertheless, the precise characterization of coagulation changes associated with early mortality remains elusive. We examined alterations in indicators linked to mortality outcomes at both the transcriptomic and clinical characteristic levels. At the transcriptomic level, we pinpointed 28 differentially expressed coagulation-related genes (DECRGs) following burn injuries and endeavored to validate their causal relationships through Mendelian randomization. DECRGs tied to survival exhibit a significant association with neutrophil function, wherein the expression of CYP4F2 and P2RX1 serves as robust predictors of fatal outcomes. In terms of clinical indicators, early levels of D-dimer and alterations in serum calcium show a strong correlation with mortality outcomes. Coagulation depletion and fibrinolytic activation, stemming from the hyperactivation of coagulation pathways post-severe burns, are strongly linked to patient mortality. Monitoring these early coagulation markers with predictive value can effectively identify individuals necessitating priority critical care.

## Introduction

Severe burns trigger a cascade of systemic responses within the body, involving the activation of multiple physiological systems. These responses encompass significant early alterations in both the coagulation and fibrinolytic systems^1^. Early activation of coagulation not only promotes hemostasis within the locally impaired microcirculation, but is also appears to contribute to the mitigation of inflammatory processes^2^. However, the intricate interplay between coagulation, fibrinolysis, the complement system, and immuno-inflammation in vivo often leads to the overactivation of coagulation and, in extreme cases, the development of disseminated intravascular coagulation (DIC)^3–5^. A critical aspect to consider is that current evidence suggests that the presence of early coagulation disorders subsequent to severe burns constitutes a substantial risk factor for a range of adverse outcomes, including death^6^. Moreover, the coagulation changes identified by previous studies that are associated with mortality outcomes remain highly controversial^7^, and only a limited number of studies have leveraged early coagulation indicators for the prediction of mortality outcomes.

**Figure 1:**
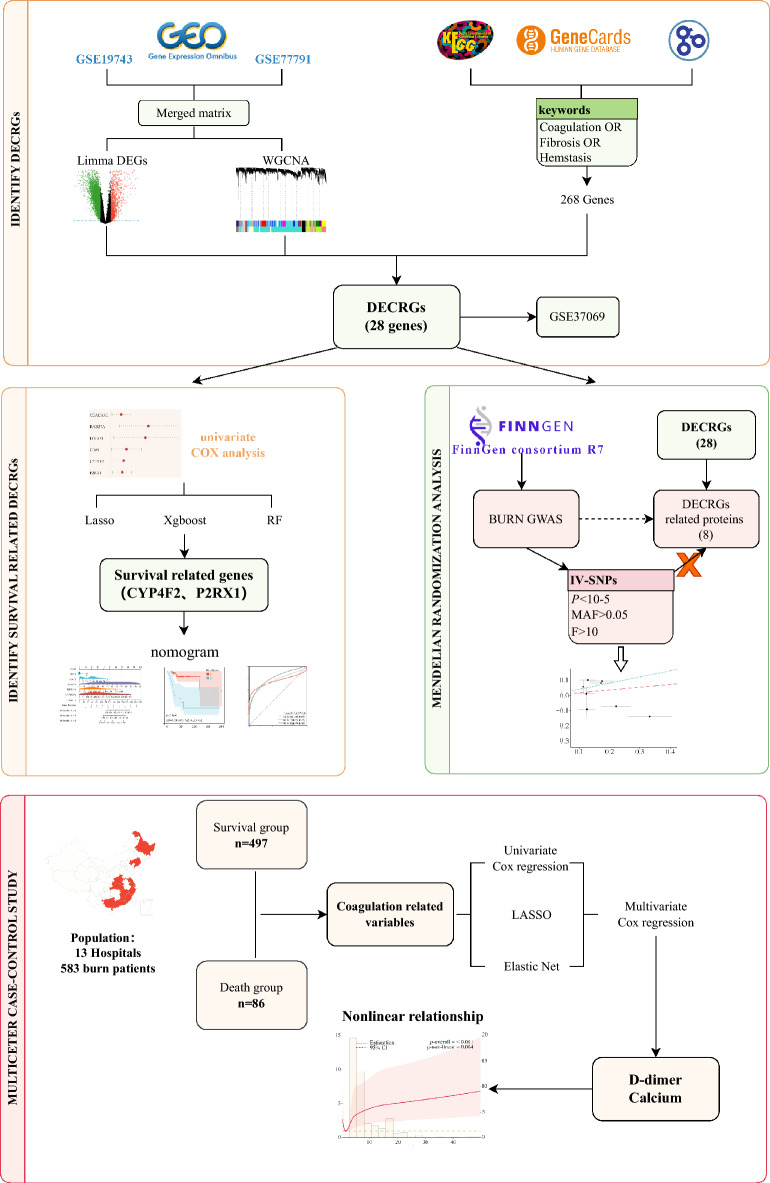
Flowchart of this study.

The primary objective was to pinpoint coagulation indicators that significantly contribute to the risk of mortality on severe burns patients from the perspective of transcriptomic and clinical characterization, respectively. And then explore whether there is a common point for survival related coagulation alterations with the combination of the two perspectives. We conducted an analysis within the Gene Expression Omnibus (GEO) database, utilizing bioinformatics methods to identify differentially expressed genes related to coagulation post-burn injury, referred to as differentially expressed coagulation-related genes (DECRGs). Subsequently, we proceeded to identify core DECRGs that exhibit robust associations with mortality outcomes. Furthermore, to ascertain the potential causality, we employed Mendelian randomization analysis to investigate the causal links between plasma DECRGs related proteins and burn injury. Finally, our investigation entailed a retrospective analysis of early clinical data drawn from twelve medical centers nationwide and assessed clinical coagulation indicators associated with mortality outcomes (Fig. 1). Our findings provide critical clinical evidence that underscores the significance of early coagulation alterations in the aftermath of burn injuries.

## Result

### Identification survival related DECRGs

The transcriptomic cohort included a total of 72 burn patients with an overall burn area of 67% from the GEO database, of which 75% were male patients. The cases of deaths were 17 (23.61%) and age and TBSA differed between the dead and surviving groups (Table 1).

**Table 1:** Patient demographics and baseline characteristics of transcriptomic cohort.

Characteristic	Overall, n = 72^1^	Survival, n = 55^1^	Death, n = 17^1^	*P* value^2^
Sex-male	54 (75%)	44 (80%)	10 (59%)	0.109
Age (year)	46 (27, 55)	39 (21, 51)	56 (48, 64)	0.003
TBSA (%)	67 (51, 78)	63 (48, 77)	77 (62, 90)	0.025
Inhalation injury	37 (65%)	29 (69%)	8 (53%)	0.274
CYP4F2 level(log2)	7.53 (7.05, 8.09)	7.27 (6.68, 7.90)	7.99 (7.63, 8.29)	0.002
P2RX1 level(log2)	8.80 ± 0.73	8.69 ± 0.69	9.18 ± 0.75	0.022

^1^n (%); Median (IQR); Mean ± SD.

^2^Wilcoxon rank sum test; Welch Two Sample t-test; Fisher’s exact test; Pearson’s Chi-squared test.

*TBSA* total body surface area.

**Figure 2:**
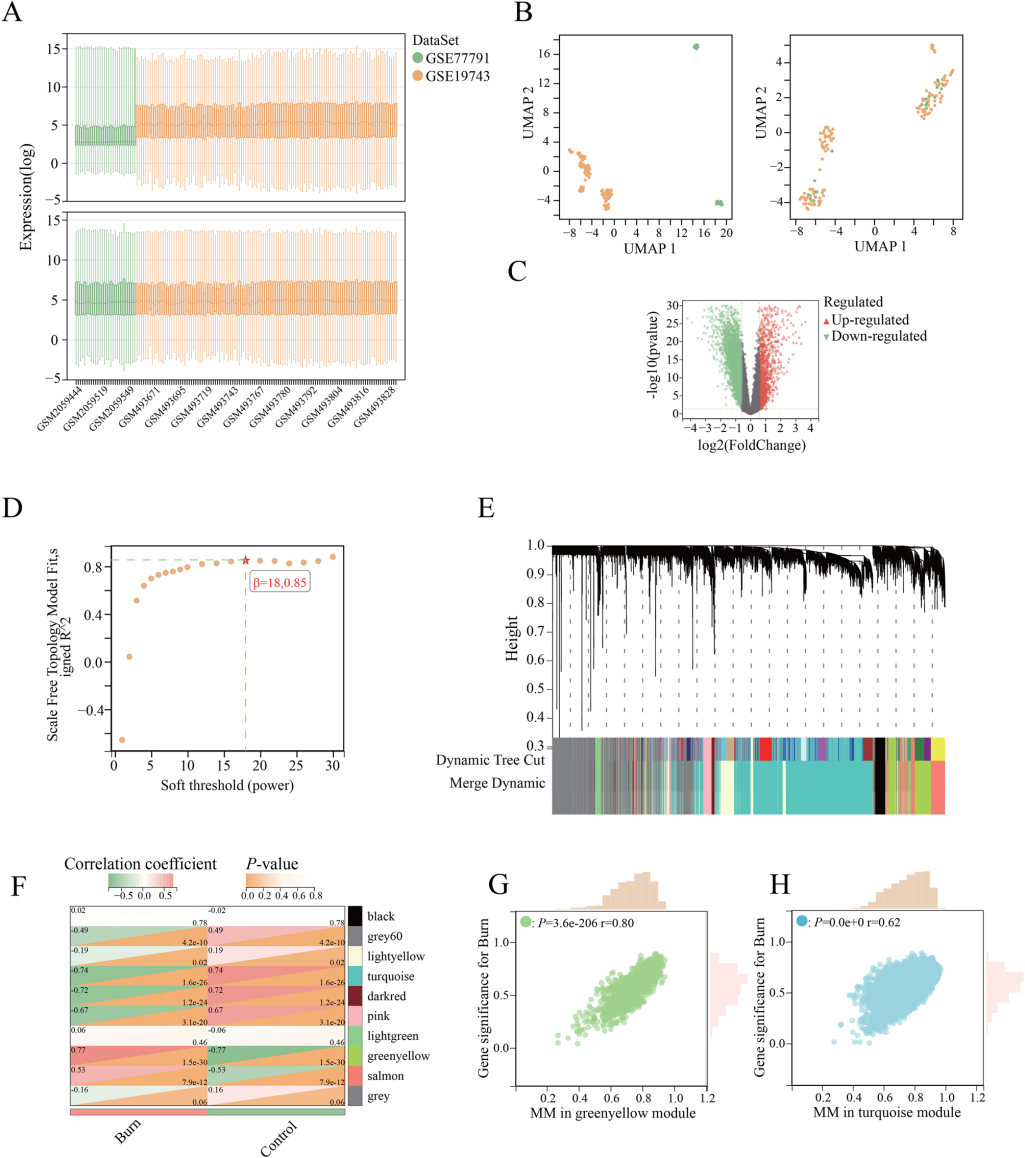
Identification of differentially expressed genes after burn injury. (**A**): Relative expression between datasets before and after removal of batch effects; (**B**): Distribution between data sets before and after removal of batch effects; (**C**) Volcano map of differentially expressed genes; (**D**): WGCNA threshold determination; (**E**): Cluster analysis of co-expression modules; (**F**): Correlation of co-expression modules with burn injuries; (**G**): Scatterplot of module membership versus gene significance for burn in greenyellow module; (**H**): Scatterplot of module membership versus gene significance for burn in turquoise module. *MM* module membership.

**Figure 3:**
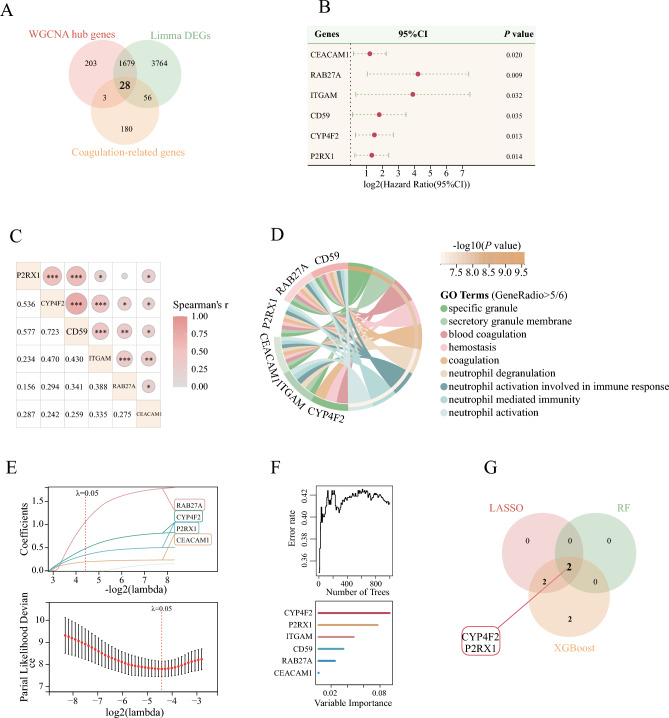
Identification of survival related DECRGs. (**A**): DECRGs identified by DEGs and WGCNA jointly; (**B**): Forest plot of the hazard ratio after univariate Cox regression; (**C**): Correlation between survival related DECRGs; (**D**): GO annotation of survival-related genes; (**E**): LASSO algorithm screens for survival related signature genes; (**F**): Random Forest algorithm screens for survival related signature genes; (**G**): Survival related DECRGs identified by all three algorithms. *WGCNA* Weighted correlation network analysis, *DECRGs* differentially expressed coagulation-related genes, *DEGs* Differential expression genes, *RF* Random Forest, *LASSO* least absolute shrinkage and selection operator.

We conducted DEGs analysis and WGCNA respectively on two merged transcriptomic datasets obtained from the GEO database. Our analysis resulted in the identification of 5527 DEGs. In the WGCNA analysis, we ultimately identified 10 co-expression modules (Fig. 2A–E). Notably, the greenyellow and turquoise modules exhibited the strongest correlation with burn, and we extracted a total of 1913 core genes from these modules (Fig. 2F–H). Coagulation-related genes were extracted from those co-occurring in DEGs and core genes, resulting in a set of 28 DECRGs (Fig. 3A, Table S2 in supplementary). To validate our findings, we assessed the expression of these 28 DECRGs in an additional dataset, GSE37069. The results showed that all were differentially expressed (Fig. S1 in supplementary).

**Figure 4:**
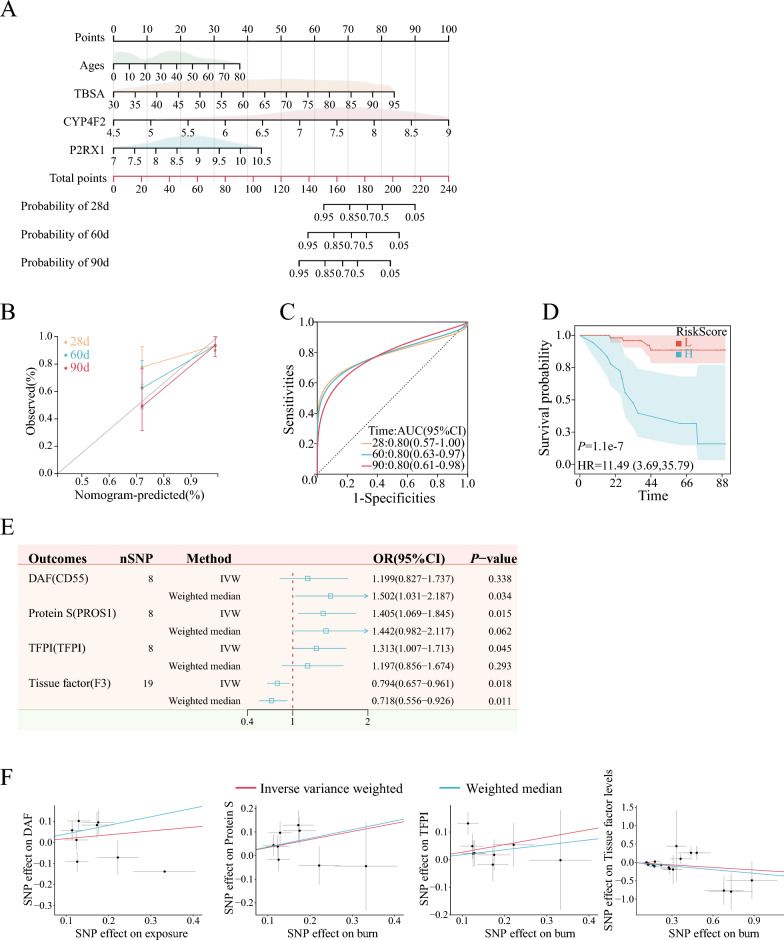
Survival-related gene prediction models and causal validation of MR. (**A**): Nomogram Incorporating Characterized Genes Predicts Risk of Death; (**B**): Calibration curve between observed and predicted outcome for nomogram; (**C**): ROC Comparison of the nomogram model for Survival Prediction; (**D**): Survival curves divided by risks core in the nomogram model; (**E**): Forest plot of positive results of MR analysis; (**F**): Scatterplot of MR positive results. *TBSA* total body surface area, *HR* Hazard Ratio, *SNP* single nucleotide polymorphism, *DAF* decay accelerating factor, *TFPI* tissue factor pathway inhibitor, *IVW* inverse variance weighted.

Further univariate Cox regression analysis of the 28 genes identified 6 genes that showed potential associations with survival outcomes (Fig. 3B). Subsequent correlation analysis revealed a significant phase relationship between P2RX1, CYP4F2, and CD59 (Fig. 3C). GO annotation of the six genes screened revealed that these genes are involved in neutrophil activation and function, where minimum set of genes was set to be 5. Building upon this finding, we employed three algorithms, namely LASSO, Random Forest, and XGBoost, to identify genes associated with the outcome. Ultimately, CYP4F2 and P2RX1 emerged as the genes associated with survival outcomes, as consistently identified by all three algorithms (Fig. 3D–G, Table 1, and Table S3 in supplementary). In our nomogram prediction model, we incorporated four variables: age, TBSA, CYP4F2 level, and P2RX1 level. The prediction model yielded a C-index of 0.818 with a 95% CI ranging from 0.680 to 0.957 and a *P* value of 6.58 × 10^–06^. Furthermore, the calibration curve demonstrated consistency between predicted and observed values. The model achieved an area under the curve (AUC) value of 0.80 for predicting outcomes at 28, 60, and 90 days. Risk scores were stratified based on the model’s criteria, with the optimal cutoff value determined as 1.728 using the R package maxstat. The significance of survival differences between subgroups, assessed via the Logrank test method, yielded a *P* value of 6.40 × 10^–8^ in survival curves (Fig. 4A–D).

### Causal relationship between burns and expression of DECRGs

Given the intricate regulation of the in vivo coagulation system, we proceeded to investigate causal alterations in DECRGs related proteins following burn injuries using MR analysis. Among the 28 DECRGs, we conducted GWAS for plasma proteins regulated by 13 of these genes. Utilizing the IVW method, we identified a potential causal relationship between burn exposure and protein S, tissue factor pathway inhibitor (TFPI), and tissue factor (TF) (Fig. 4E). Simultaneously, the Weighted mode method identified decay accelerating factor (DAF) and TF. Notably, DAF, protein S, and TFPI exhibited positive directional trends, while TF displayed a negative directional trend (Fig. 4F). Importantly, none of the aforementioned positive results exhibited horizontal pleiotropy or heterogeneity following sensitivity analysis, except for DAF (*P* = 0.049) (Table S4 in supplementary).

### D-dimer and serum calcium as risk factors for death

In this retrospective clinical cohort, we enrolled 583 patients with severe burns, among whom 86 (14.75%) unfortunately succumbed to their injuries. The overall male proportion of the cohort was 70%, the median age was 48 years, and the median burn area was approximately 60%. There were no statistically significant differences in gender, age, TBSA, incidence of inhalation injuries, or mortality between retrospective cohort and transcriptomic cohort (Table S5 in supplementary).

Regarding coagulation indicators, except thrombin time and fibrinogen, notable differences were observed between the groups. The death group exhibited prolonged prothrombin time (PT) and activated partial thromboplastin time (APTT), along with increased levels of D-dimer, INR, and platelets. The death group displayed a decrease in pH and albumin levels, along with an increase in total bilirubin (TLIB) and alkaline phosphatase. Statistically significant differences were not observed in other parameters, including the cause of injury, medical history, and the use of anticoagulant therapy (Table 2).

**Table 2:** Patient demographics and baseline characteristics of retrospective cohort.

Characteristic	Overall, n = 583^1^	Survival, n = 497^1^	Death, n = 86^1^	*P*-value^2^
Sex-male	409 (70%)	345 (69%)	64 (74%)	0.349
Age (year)	48 (36.00, 57.50)	47 (35.00, 56.00)	56 (47.25, 68.00)	< 0.001
TBSA (%)	60.0 (44.50, 80.00)	55.0 (40.00, 75.00)	82.5 (61.25, 94.00)	< 0.001
Weight (kg)	65.0 (58.00, 72.25)	65.0 (58.00, 72.00)	65.0 (60.00, 73.50)	0.459
Cause				0.294
Fire	479 (82%)	404 (81%)	75 (87%)	
Corrosive injury	19 (3%)	18 (4%)	1 (1%)	
Scald injury	59 (10%)	50 (10%)	9 (10%)	
Electric injury	26 (4%)	25 (5%)	1 (1%)	
Inhalation injury	433 (74%)	377 (76%)	56 (65%)	0.035
Coagulation function				
Prothrombin time (s)	11.7 (11.00, 12.70)	11.6 (11.00, 12.60)	12.3 (11.30, 14.00)	0.002
APTT (s)	27.2 (23.50, 32.50)	27.0 (23.30, 31.60)	31.6 (24.63, 37.80)	0.003
Thrombin time (s)	18.5 (16.90, 20.20)	18.5 (16.90, 20.10)	18.6 (16.63, 20.28)	0.709
D-dimer (mg/L)	2.6 (1.12, 5.94)	2.3 (1.04, 5.25)	5.3 (2.68, 15.32)	< 0.001
INR	1.0 (0.93, 1.10)	1.0 (0.93, 1.08)	1.1 (0.97, 1.20)	< 0.001
Fibrinogen (g/L)	2.5 (1.98, 3.17)	2.5 (2.00, 3.12)	2.6 (1.94, 3.36)	0.626
Calcium (mmol/L)	2.0 (1.82, 2.19)	2.0 (1.82, 2.19)	2.0 (1.82, 2.17)	0.837
PH	7.4 (7.33, 7.42)	7.4 (7.33, 7.42)	7.4 (7.30, 7.40)	0.034
Lactic acid (mmol/L)	3.8 (2.30, 5.25)	3.5 (2.20, 5.15)	4.5 (3.16, 5.68)	0.001
Liver and kidney function				
Total bilirubin (μmol/L)	17.1 (11.50, 25.25)	16.3 (11.40, 24.20)	20.5 (12.85, 30.53)	0.010
Creatinine (μmol/L)	74.0 (58.00, 93.05)	70.2 (56.00, 88.00)	97.7 (79.63, 147.83)	< 0.001
Alkaline phosphatase (U/L)	61.9 (51.00, 72.42)	61.0 (51.00, 72.00)	62.9 (52.25, 73.75)	0.345
Albumin (g/dL)	33.3 (27.30, 38.30)	33.7 (28.30, 38.30)	28.7 (23.33, 36.95)	0.001
Blood cell parameters				
RBC	5.3 (4.79, 5.83)	5.3 (4.79, 5.83)	5.2 (4.75, 5.77)	0.418
WBC	19.9 (14.38, 26.54)	19.2 (14.03, 26.00)	23.3 (16.87, 29.88)	0.002
Platelet (10^9^/L)	238.0 (178.00, 309.50)	234.0 (172.00, 304.00)	264.5 (197.25, 362.50)	0.016
Hemoglobin level (g/L)	161.0 (143.00, 177.00)	161.0 (144.00, 176.00)	164.0 (141.25, 180.75)	0.839
Time post burn (hours)	3.0 (2.00, 6.00)	3.7 (2.00, 6.00)	3.0 (2.00, 5.00)	0.131
Length of stay (days)	36.0 (21.00, 62.00)	40.0 (27.00, 66.00)	9.0 (4.00, 18.00)	< 0.001
Anamnesis				
Hypertension	49 (8%)	38 (8%)	11 (13%)	0.112
Diabetes mellitus	19 (3%)	15 (3%)	4 (5%)	0.505
Chronic cardiac disease	7 (1%)	5 (1%)	2 (2%)	0.276
Stroke	9 (2%)	6 (1%)	3 (3%)	0.134
Intervention				
CRRT	21 (4%)	9 (2%)	12 (14%)	< 0.001
Anticoagulation	68 (12%)	57 (11%)	11 (13%)	0.724
Mechanical ventilation	225 (39%)	171 (34%)	54 (63%)	< 0.001

^1^n (%); median (IQR).

^2^Pearson’s Chi-squared test; Wilcoxon rank sum test; Fisher’s exact test.

*TBSA* total body surface area, *APTT* activated partial thromboplastin time, *INR* international normalized ratio, *TLIB* total bilirubin, *RBC* red blood cell, *WBC* white blood cell, *CRRT* continuous renal replacement therapy.

**Figure 5:**
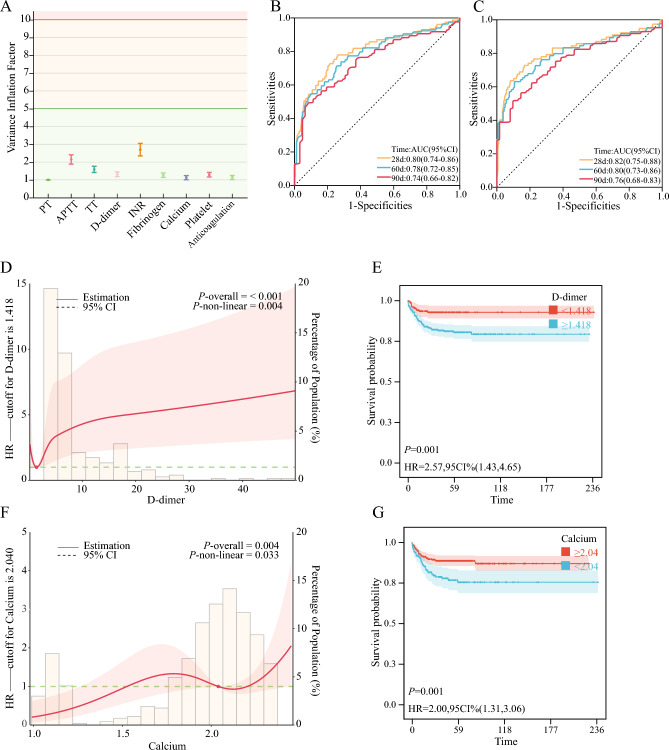
D-dimer and serum calcium are risk factors for death. (**A**): VIF values after multicollinearity test for variables. (**B**): ROC Comparison of Age and TBSA for Survival Prediction. (**C**): ROC Comparison of Age, TBSA, D-dimer, and calcium for Survival Prediction. (**D**): Restricted cubic spline for D-dimer and survival outcomes. (**E**): Survival curves divided by D-dimer cutoffs. (**F**): Restricted cubic spline for calcium and survival outcomes. (**G**): Survival curves divided by calcium cutoffs. *TBSA* total body surface area, *APTT* activated partial thromboplastin time, *INR* international normalized ratio, *AUC* area under curve, *HR* hazard ratio, *CI* confidence interval.

For further analysis of influential factors, we incorporated coagulation-related indicators and assessed them for multicollinearity. The VIF for all variables was found to be less than 5 (Fig. 5A). Through univariate and multivariate Cox regression analyses, we identified D-dimer (HR 1.01, 95% CI 1.00–1.02) and calcium (HR 4.01, 95% CI 1.82–8.83) as associated with mortality outcomes, establishing them as independent risk factors. Acknowledging the existence of inevitable interactions (Fig. S2 in supplementary), we proceeded to screen the variables using elastic networks and the LASSO algorithm. The outcome remained significant (*P* < 0.05) (Table 3, Table S6 in supplementary). The inclusion of D-dimer and calcium in the survival outcome prediction model resulted in enhanced prediction accuracy across multiple time intervals: 28 days, 60 days, and 90 days (Fig. 5B,C). Mediation analyses revealed that the level of D-dimer appeared to mediate the effect of TBSA on mortality outcomes (Table S7 in supplementary).

**Table 3:** Univariate and multivariate analysis of risk factors in retrospective cohort (Cox regression).

Methods	D-dimer		Calcium	
	HR (95%CI)	*P* value	HR (95%CI)	*P* value
Univariable	1.02 (1.01–1.03)	< 0.001	1.57 (0.88–2.78)	0.125
Model1	1.01 (1.00–1.02)	0.02	2.66 (1.40–5.04)	0.003
Model2	1.01 (1.00–1.02)	0.049	3.92 (1.83–8.38)	< 0.001
LASSO	1.02 (1.01–1.03)	0.001	2.98 (1.43–6.23)	0.004
Stepwise-backward	1.01 (1.00–1.02)	0.038	3.83 (1.79–8.19)	0.001
Elastic Net	1.02 (1.01–1.03)	0.001	2.95 (1.41–6.17)	0.004

HR = Hazard Ratio, CI = Confidence Interval. Model1: adjusted for age, TBSA and inhalation injury; Model2: adjusted for age, TBSA, inhalation injury, PH, lactic acid, total bilirubin, creatinine, albumin and WBC.

### Nonlinear relationship between D-dimer, calcium and mortality outcome

In clinical practice, the relationship between independent and dependent variables is frequently nonlinear, particularly when the independent variable is continuous. Categorizing continuous variables based on arbitrary truncation values can lead to missing data. First of all, the Schoenfeld test proves that the Cox model meets the proportional risk assumption (Fig. S3 in supplementary). Furthermore, we observed that age and TBSA were independent risk factors for mortality. RCS analysis, adjusted for covariates age and TBSA, revealed a nonlinear dose–response relationship between D-dimer levels and the risk of death (*P* < 0.05), with a cutoff point at 1.418 mg/L (Fig. 5D). When D-dimer levels exceeded 1.418 mg/L, an incremental increase in D-dimer was associated with a progressively elevated risk of death. Segmented regression analysis at this cutoff point demonstrated that D-dimer levels exceeding 1.418 mg/L were statistically significant as a risk factor (HR 1.02, 95% CI 1.01–1.03, *P* < 0.001), whereas levels below 1.418 mg/L were not statistically significant. Likewise (Table 4), RCS analysis revealed a nonlinear association between calcium levels and mortality outcomes, with calcium levels below 2.04 mmol/L being a risk factor for death (HR 3.53, 95% CI 1.38–9.04, *P* = 0.008) (Fig. 5E, Table 4). In survival curves, it is evident that D-dimer and calcium, stratified by the cutoff values, effectively differentiate survival risk (Fig. 5F,G).

**Table 4:** Effect of d-dimer and calcium level on survival: adjusted hazard ratios from segmented cox regression analysis.

Characteristic	HR	95% CI	*P*-value
D-dimer (mg/L)			
< 1.418	0.45	0.10, 1.97	0.290
≥ 1.418	1.02	1.01, 1.03	< 0.001
Calcium (mmol/L)			
< 2.04	3.53	1.38, 9.04	0.008
≥ 2.04	2.5	0.24, 26.4	0.450

*HR* Hazard Ratio, *CI* Confidence Interval. *HRs* were adjusted for TBSA and Age.

## Discussion

Previous research has indicated that coagulation disturbances exhibit dynamic changes throughout the progression of burn injuries^7^. Early alterations in the coagulation system may be less discernible in patients with smaller burn areas. However, in patients with extensive burns, these changes can manifest within 24 h of injury. The precise pathophysiology underlying early coagulation changes in severe burn patients remains uncertain, with endothelial injury and tissue hypoperfusion currently the main thought drivers^8^. Extensive vascular endothelial damage and capillary leakage lead to a self-protective activation of coagulation pathways within the body. Damage-associated molecular patterns (DAMPs), molecular fragments generated in stressed or damaged tissues following burn injuries, are detected by pattern recognition receptors on the surfaces of a wide array of cells throughout the body^9^. The binding of DAMPs not only induces TF expression and activates external coagulation pathways but also triggers complement cross-linking with the coagulation system^10^. Within the context of systemic multisystem crosstalk, coagulation exhibits significant heterogeneity.

Due to the limited specificity, we proceeded to identify DECRGs with potential effects on survival outcomes at the transcriptomic level. These 28 DECRGs encompass regulators and proteins associated with diverse coagulation pathways, encompassing the complement system, hemostatic processes, and fibrinolysis. GO analysis of the six DECRGs identified as survival-related through univariate Cox regression unveiled a predominant involvement of neutrophils and their associated functional components. In the setting of widespread activation of coagulation and immunoinflammatory systems following burn injuries in vivo, DAMPs induced early in the course of burn injuries trigger the expression of TF on the surface of innate immune cells and activate external coagulation pathways^11^. Neutrophils, as pivotal components of the innate immune system, serve as essential mediators and play a significant role in fibrin formation during the early post-burn period^12^. In a laser-induced vascular endothelial injury model, neutrophils are the initial cells to accumulate within the vessel wall, interact with and aggregate around damaged endothelial cells, and contribute to thrombus formation^13^. Importantly, DAMPs also stimulate neutrophil degranulation, leading to the release of serine proteases (such as elastase and histone G) and the formation of neutrophil extracellular traps^14^. Previous research has primarily associated the impact of neutrophil serine proteases on the coagulation process with their role in promoting the inactivation of TFPI and fibrin production^12^. Early activation of neutrophils contributes to hemostasis and local inflammatory responses, but excessive dysregulation can result in coagulation depletion and a systemic immune-inflammatory response that is more likely to lead to adverse outcomes.

The expression of genes associated with fatal outcomes, namely P2RX1 and CYP4F2, seems to contribute to the process of neutrophil hyperactivation. P2X1 receptors, which are ATP-gated ion channels, are expressed on both platelets and neutrophils^15,16^. ATP ligands increase in the extracellular space of the endothelium following tissue injury. This channel exhibits high permeability to Ca^2+^, with approximately 10% of the action potential mediated by these ions^17^. In platelets, P2X1 receptors mediates the activation of ERK2 phosphorylation via increased intracellular Ca^2+^, thereby enhancing the aggregation response to thrombin^18^. Additionally, the accumulation and pro-thrombotic capacity of neutrophils rely on the P2X1 receptor. Mice lacking the P2X1 receptor exhibited a substantial reduction in neutrophil chemotaxis and fibronectin production at the site of endothelial injury when compared to wild mice^15^. CYP4F2 serves as a vitamin K1 oxidase and an ω-hydroxylase of menaquinone 4 (MK-4)^19,20^. Moreover, genetic polymorphisms in the CYP4F2 gene impact plasma concentrations of vitamin K1 and MK-4. Vitamin K, functioning as a cofactor for gamma-glutamyl carboxylase, plays a crucial role in the hepatic metabolism of proteins C and S, along with the synthesis of coagulation factors II, VII, IX, and X^21^. Furthermore, genetic polymorphisms in CYP4F2 are linked to the necessary dosage of warfarin^22^.

Following MR analysis, it was observed that plasma TF levels decreased after exposure to burns, while there was an increase in the expression of negatively regulated factors such as TFPI, protein S, and DAF. TFPI plays a pivotal role as a major inhibitor of the FVIIa-TF complex within the exogenous coagulation pathway, and protein S functions as an inhibitory cofactor that enhances the inhibitory capacity of TFPI^1^. DAF serves as a negative regulator within the complement cascade^23^. In summary, following burn injuries, genetic variation perspectives indicate a somewhat heightened activation of negative regulatory mechanisms. This aligns with findings from previous studies focusing on early trauma^24^; however, further examination is required within the context of burn patients. Given the dynamic nature of burn injuries, the insights we have presented may necessitate additional validation through rigorous animal or clinical trials.

It has been proposed that early changes in severe burns involve the activation of coagulation pathways, a decrease in natural anticoagulants (protein C and protein S), and the initiation of fibrinolysis^1^. Nonetheless, some scholars contend that coagulation experiences heightened activation, leading to microvascular thrombosis subsequent to coagulation dysregulation caused by consumptive coagulation disorders^25^. This results in a state of hypocoagulability and increased fibrinolysis. In our study, we observed prolonged PT and APTT in the death group, supporting the notion that early hypocoagulation may hold predictive value for survival outcomes.

Importantly, early fibrinolytic activation has been consistently observed in the majority of studies, and it represents a significant factor contributing to mortality in trauma patients, including those with burns^26–29^. This phenomenon may be attributed to the release of substantial levels of free tissue-type plasminogen activator from damaged endothelial cells, ultimately resulting in an upregulation of fibrinogenesis^30^. In our multicenter population-based study, D-dimer, a marker reflecting activation of fibrinogenesis, was significantly elevated in the death group and was an independent predictor of survival outcome. Previous investigations have demonstrated that D-dimer levels are elevated upon admission in burn patients^25^. Moreover, early elevation in D-dimer levels in trauma patients is believed to correlate not only with the severity of injury but also to predict multiple adverse outcomes, including hemorrhage, disseminated intravascular coagulation (DIC), and death^31^. In severely traumatized patients, Hayakawa et al. identified an optimal cutoff value of 38 mg/L for 24-h D-dimer levels associated with mortality outcomes using receiver operating characteristic (ROC) analysis^28^. In our study, we observed an association between D-dimer levels exceeding 1.418 mg/L and mortality outcomes, with a corresponding increase in the risk of death as D-dimer levels rose. Given that endothelial injury, tissue hypoperfusion, and systemic inflammation frequently co-occur in severe burns, a mild fibrinolytic status may serve as a predictive marker for adverse outcomes.

Besides D-dimer, serum calcium levels below 2.03 mmol/L were also linked to an elevated risk of mortality. As a pivotal element of P2X1 receptors in the coagulation pathway, excessive depletion of blood calcium independently contributes to mortality risk. In addition, neutrophil activation after injury leads to increased intracellular calcium mobilization^32^. While previous studies have observed a decrease in serum calcium levels following trauma, whether it is associated with adverse outcomes is inconsistent among different studies^33,34^. Likewise, in our study, we initially did not observe any disparity in calcium levels between the groups that survived and those that did not. Furthermore, no statistical significance was evident in the univariate Cox analysis. However, positive findings surfaced following variable screening. This may warrant further consideration of the interaction of serum calcium with other factors, which is also more reflective of the real-world impact of serum calcium levels.

When combined with transcriptomic and clinical characteristics analysis, it was observed that patients in the death group exhibited elevated levels of both fibrinolysis and thrombosis. Furthermore, this phenomenon is associated with the early activation of neutrophils. Based on this observation, we formulated a hypothesis that adverse outcomes may be linked to early secondary coagulation disorders arising from hyperactivation of the coagulation system. Excessive coagulation consumption could potentially manifest as the depletion of various factors. However, it is important to note that the present study did not comprehensively and meticulously collect indicators such as coagulation factors and fibrinolysis from a large number of patients. This is an aspect that we intend to address more rigorously in our future investigations. Given the intricate interplay with various immune-inflammatory systems, it appears challenging to intervene directly in the coagulation pathway solely based on alterations in patient coagulation screening indicators. Furthermore, it is worth noting that extensive assessments of thrombosis and hemostasis have not yet been undertaken at the clinical level. Utilizing four valuable predictive indicators allows for the identification of individuals at risk, thereby enabling the implementation of additional interventions and tailored care for this population. The identification of survival-related transcriptomic genes and clinical characteristics is based on different population cohorts. Some valuables such as age, TBSA, and the incidence of inhalation injury are thought to be strongly associated with mortality in previous studies. No statistically significant differences were observed in the age, TBSA, and incidence of inhalation injury in the populations included in our study. And the mortality rates among populations are similar, so we assumed that they are still comparable.

There are also some limitations in this paper. Firstly, we encountered limitations in obtaining GWAS data for all DECRGs candidates, and our MR analysis was consequently restricted to the available metrics. Secondly, our study did not involve the analysis of variations in mRNA and protein expression levels of the core genes through cellular experiments. Thirdly, the current lack of open studies of burned patients still exists, and thus genetic diversity and potential differences between the different population cohorts in our study are still not well avoided. However, we provide a method of analysis that may be flawed with the current data, but also provides direction for future research. Lastly, we could only gather information regarding the patients’ prior medical histories, particularly concerning coagulation deficiencies and pre-hospitalization anticoagulant medications, through verbal inquiries.

## Methods

### Bioinformatics analysis

To identify DECRGs, we initiated our analysis from a bioinformatic perspective. Burn-related blood transcriptome sequencing data were retrieved from two GEO databases (http://www.ncbi.nlm.nih.gov/geo/): GSE77791 (burn patients = 15, control = 13) and GSE19743 (burn patients = 57, control = 63). To ensure consistency, we selected the earliest available sequencing data from each patient, acquired within 24 h post-injury, for further analysis. Genes associated with the human coagulation pathway were collected from three reputable databases: KEGG, AmiGo^35^, and GeneCards^36^. The search keywords employed included "coagulation", "fibrinolysis", and "hemostasis". We further refined our gene selection by including 268 genes that were concurrently present in two or more of these databases.

Initially, we merged the two datasets employing the R package inSilicoMerging^37^. Subsequently, to eliminate any potential batch effects, we applied the methodology outlined by Johnson et al^38^. Differential expression genes (DEGs) analysis was conducted using the R package limma after log2 transformation of the data^39^. Genes were deemed differentially expressed if they exhibited an adjusted *P* value of less than 0.05 and an absolute fold change greater than 1.5.

Weighted correlation network analysis (WGCNA) is a robust systems biology method employed to elucidate gene association patterns among samples^40^. It enables the identification of highly synergistic gene sets and the exploration of candidate biomarker genes or therapeutic targets by integrating gene-set integration and gene-set-phenotype associations. In our analysis, we selected a power threshold of *R*^2^ = 0.85 and a soft threshold of *β* = 18. Gene significance was utilized to measure the correlation between genes and clinical phenotypes, while module membership captured the correlation between eigengene and gene expression profiles of modules. We identified core genes as those within modules demonstrating both clear significance and a high correlation with burn injury, with a module membership threshold exceeding 0.8.

### Identification survival related DECRGs

Correlate limma-identified DEGs, WGCNA-identified hub genes, and coagulation-related genes with each other to identify DECRGs. Initially, we employed univariate Cox regression to identify genes potentially linked to mortality outcomes. Gene Ontology (GO) of the screened survival-associated DECRGs was performed by clusterProfiler package in R, and *P* value < 0.05 were considered statistically significant. Subsequently, we subjected the characterized genes to a comprehensive screening process. This screening involved the combination of least absolute shrinkage and selection operator (LASSO), Random Forest, and XGBoost algorithms. The LASSO regression algorithm, by adding a penalty term in the model estimation, can compress the regression coefficients of some unnecessary variables to zero and then eliminate them from the model to achieve the purpose of variable screening^41^. The DECRGs that emerged as survival-related were harnessed for survival prediction analysis. Finally, the expression of DECRGs was validated in an additional dataset GSE37069.

### Mendelian randomization analysis

Changes following burn injuries are intricate, involving multiple systems and levels. Traditional methods often struggle to uncover the specific causal links between burn injuries and alterations in various coagulation indicators. To address this challenge, we employed an innovative epidemiological approach known as Mendelian randomization (MR) analysis^42^. MR analysis uses genetic variation as an instrumental variable to investigate causal relationships between exposures and outcomes^43^. In this study, we conducted a two-sample MR analysis with burn injury as the exposure factor and DECRGs related proteins as the outcome factor. Burn-related Genome-Wide Association Study (GWAS) data were acquired from the FinnGen database, encompassing 3134 cases and 306,020 controls. GWAS data for DECRGs related proteins in plasma were obtained from previous studies^44–46^. The populations included in these datasets were all of European origin (Table S1 in supplementary).

The significance threshold for instrumental variable screening was 1 × 10^–5^, considering that instrumental variables (single nucleotide polymorphism) require strong correlation with exposure factors but not with outcome factors^47^. We employed two distinct MR analysis methods, Inverse Variance Weighted (IVW) and Weighted Median, to assess the specific association of genetically predicted burns with DECRGs related proteins. In addition, we conducted a sensitivity analysis for the MR results by applying Cochran’s Q-test to assess the heterogeneity between instrumental variables, and a *P* value less than 0.05 indicated the presence of heterogeneity. In cases where heterogeneity was detected, we employed MR Egger regression to further eliminate the potential effect of horizontal pleiotropy. Once again, a *P* value less than 0.05 indicated the presence of heterogeneity that required correction^48^.

### Analysis of clinical coagulation indicator influencing survival outcomes

We recruited patients with burn injuries meeting the criteria of either a total body surface area (TBSA) of ≥ 30% burns or ≥ 10% third-degree burns. These patients were admitted to the hospital within 24 h post-injury and completed relevant clinical laboratory tests from twelve prominent tertiary hospitals across mainland China during the period spanning January 01, 2015, to December 30, 2020. The coagulation indicators of the patients were assayed by the professionals of the laboratory department of the medical center. Patients’ venous blood was collected with 5 ml of citrate-containing blood collection tubes and sent for testing within 1 h at room temperature after collection. Whole blood was centrifuged to obtain plasma, which was further analyzed using a fully automated blood coagulation analyzer (Mindray CX-9000, China).

To assess the influence of early coagulation indicators on survival outcomes, we employed the Cox proportional-hazards model to identify potentially relevant variables. We also utilized the variance inflation factor (VIF) to evaluate potential multicollinearity among the variables^49^. The VIF is calculated using the formula: *VIF* = 1*/*(1* − R*^2^)*,* where *R*^*2*^ represents the coefficient of determination used by the other predictors in the model to predict the predictor to be evaluated. Each independent variable is assigned a VIF score. A VIF less than 5 is indicative of low multicollinearity, while a score falling between 5 and 10 suggests the presence of some multicollinearity. A VIF exceeding 10 indicates severe multicollinearity. Variables identified through this initial screening process were subsequently subjected to multivariate Cox regression analysis. To identify variables suitable for multifactorial analyses, we employed a systematic approach involving three main methods: (1) Variables that exhibited positive statistical significance following univariate Cox regression analysis were selected for further consideration. (2) In view of the small number of variables, we included all variables in the Multivariate analysis. (3) To address the potential impact of multicollinearity, we employed both LASSO and Elastic Net models to identify suitable variables for multifactorial analysis. The Elastic Network algorithm combines the strengths of Ridge regression and Lasso regression^50^. Tenfold cross-validation was used in variable screening to select lambda values with the smallest mean square error.

### Restricted cubic spline

To investigate potential nonlinear associations between coagulation indicators and survival outcomes, we employed the restricted cubic spline (RCS) methodology^51^. A cubic spline represents a continuous, segmented cubic polynomial, where the selection of node positions and their number is done to ensure the spline function provides a smooth curve for the continuous variable X across its entire range of values. Knot value with the smallest AIC value was selected after modeling different nodes and determined the position based on the 10th and 50th percentile. Prior to the implementation of RCS, we check whether the Cox model constructed by the variables is consistent with the proportional risk assumption using the Schoenfeld test.

### Statistical analysis

In this paper, categorical variables are expressed as numbers (%), and continuous variables were presented as either mean (SD) when they adhered to a normal distribution or median (interquartile range) when their distribution deviated from normality. All statistical analyses were performed using R software. Some graphs were visualized based on the Sanger Box analysis tool^52^. A two-sided *P* value < 0.05 was considered statistically significant.

### Ethics statement

The retrospective cohort has been approved by the ethics committee of Changhai Hospital (Grant No. CHCE2022-243).

## Conclusion

This study has illuminated early coagulation disorders linked to fatal outcomes in severe burns from a multifaceted standpoint. Early coagulation depletion and fibrinolytic activation have emerged as significant associations with mortality outcomes. Furthermore, our investigation also determined the feasibility of early alterations in D-dimer, serum calcium, CYP4F2 and P2RX1 within 24 h post-injury as predictors to build a robust predictive model that may provide early clinical targets for monitoring. Further validation of the predictive value of these metrics in cohort populations is necessary.

### Supplementary Information

Supplementary Information.

## Data Availability

Burn patient transcriptome data was obtained from the GEO database (http://www.ncbi.nlm.nih.gov/geo), accession numbers GSE77791, GSE19743, and GSE37069. GWAS data was obtained from the FinnGen (https://www.finngen.fi/en), The coagulation-related gene set is derived from three major gene databases, KEGG (https://www.genome.jp/kegg/), AmiGo (https://amigo.geneontology.org/amigo/), and GeneCards (https://www.genecards.org/).
