# Supplementary material for: Identification of early coagulation changes associated with survival outcomes post severe burns from multiple perspectives
Source: Sci Rep. 2024 May 7;14:10457. doi: 10.1038/s41598-024-61194-0 (PMC11076290; doi:10.1038/s41598-024-61194-0)
Supplement: Supplementary file 1 — Supplementary Information. [file 41598_2024_61194_MOESM1_ESM.pdf]

Legend

Table S1 Obtained GWAS data for DECRs-related proteins

Table S2 Obtained differentially expressed coagulation-related genes (DECRGs)

Table S3 Univariate and multivariate analysis of risk factors in transcriptomic cohort (Cox regression)

Table S4 MR results and sensitivity analysis

Table S5 Comparison of Patients' demographics and baseline characteristics among different cohorts

Table S6 Variables screened by three algorithms

Table S7 Mediation analysis of D-dimer and calcium for the associations between TBSA and death

Figure S1. Differential expression of 28DECRGs in GSE37069.

Figure S2 Correlation between coagulation related variables.

Figure S3 Schoenfeld test for Cox proportional-hazards model

Table S1 Obtained GWAS data for DECRs-related proteins

Genes	Protein Coding	GWAS resource				
		Year	Case	SNPs	Population	PMID
MAPK14	Mitogen-activated protein kinase 14					
CD55	DAF					
CLU	Clusterin	2017	-	501,428	European	28240269[1]
TFPI	Tissue factor pathway inhibitor					
PROS1	Protein S					
CEACAM1	Carcinoembryonic antigen-related cell adhesion molecule 1					
FYN	FYN Proto-Oncogene, Src Family Tyrosine Kinase					
PIK3R1	Phosphatidylinositol 4,5-bisphosphate 3-kinase catalytic subunit alpha isoform:Phosphatidylinositol 3-kinase regulatory subunit alpha complex	2018	3301	10,534,735	European	29875488[2]
ENTPD1	Ectonucleoside Triphosphate Diphosphohydrolase 1					
F8	Coagulation Factor VIII					
MERTK	Tyrosine-protein kinase Mer levels					
CD40LG	CD40 Ligand	2020	1301	18,166,693	European	33303764[3]
F3	Tissue factor levels					

DAF: decay accelerating factor; SNP: single nucleotide polymorphism; GWAS: Genome-Wide Association Study; PMID: PubMed ID of articles.

1. Suhre K, Arnold M, Bhagwat AM, et al. Connecting genetic risk to disease end points through the human blood plasma proteome. *Nat Commun*.

Feb 27 2017;8:14357. doi:10.1038/ncomms14357
2. Sun BB, Maranville JC, Peters JE, et al. Genomic atlas of the human plasma proteome. *Nature*. Jun 2018;558(7708):73-79. doi:10.1038/s41586-018-0175-2
3. Gilly A, Park YC, Png G, et al. Whole-genome sequencing analysis of the cardiometabolic proteome. *Nat Commun*. Dec 10 2020;11(1):6336. doi:10.1038/s41467-020-20079-2

Table S2 Obtained differentially expressed coagulation-related genes (DECRGs)

DECRGs	Coding Protein	DECRGs	Coding Protein	DECRGs	Coding Protein
CYP4F2	Mitogen-activated protein kinase 14	CEACAM1	Carcinoembryonic antigen-related cell adhesion molecule 1	MASP2	MBL Associated Serine Protease 2
TFPI	Tissue factor pathway inhibitor	RAB27A	Ras-Related Protein Rab-27A	CD40LG	CD40 Ligand
CLU	Clusterin	ITGAM	Integrin Subunit Alpha M	PIK3CB	Phosphatidylinositol-4,5-Bisphosphate 3-Kinase Catalytic Subunit Beta
MAPK14	Mitogen-activated protein kinase 14	CR1	Complement Receptor Type 1	ENTPD1	Ectonucleoside Triphosphate Diphosphohydrolase 1
MERTK	Tyrosine-protein kinase Mer levels	ITPR3	Inositol 1,4,5-Trisphosphate Receptor Type 3	F8	Coagulation Factor VIII
PIK3R1	Phosphoinositide-3-Kinase, Regulatory Subunit 1	FYN	FYN Proto-Oncogene, Src Family Tyrosine Kinase	PABPC4	Poly(A) Binding Protein Cytoplasmic 4
P2RX1	P2X1 Receptor	GNAQ	G Protein Subunit Alpha Q	AK3	Adenylate Kinase 3
PROS1	Protein S	PRKACB	Protein Kinase CAMP-Activated Catalytic Subunit Beta	F3	Tissue factor
CD59	Membrane Attack Complex Inhibition Factor	EMILIN2	Elastin Microfibril Interfacer 2		
CD55	DAF	RASGRP1	RAS Guanyl Releasing Protein 1		

DAF: decay accelerating factor

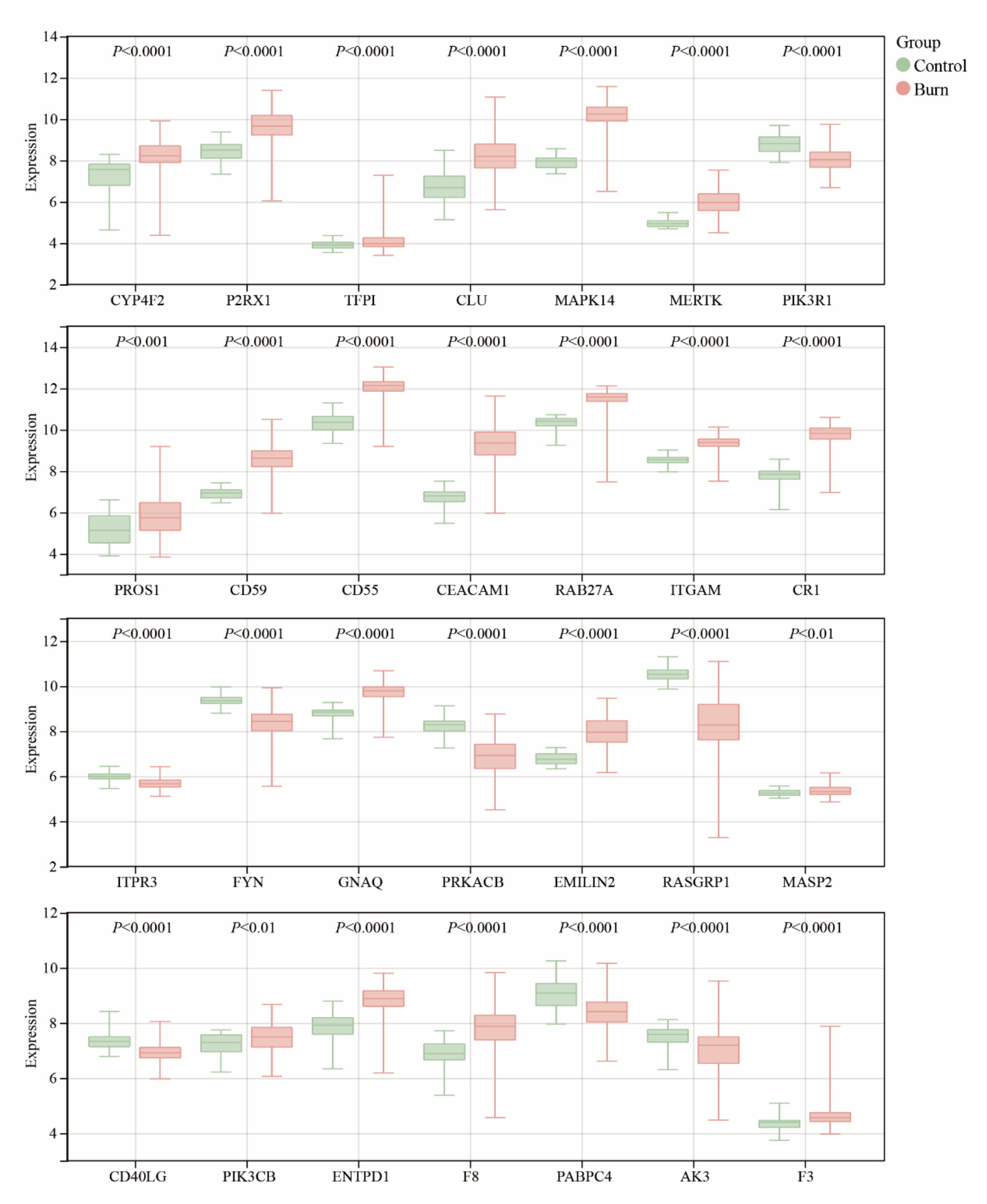

Figure S1. Differential expression of 28DECRGs in GSE37069.

Table S3 Univariate and multivariate analysis of risk factors in transcriptomic cohort (Cox regression)

Variables	Univariable		Multivariable	
	HR (95%CI)	*P* value	HR (95%CI)	*P* value
**Age**	1.03 (1.00-1.06)	0.031	1.03 (1.00-1.07)	0.056
**TBSA**	1.03 (1.00-1.06)	0.080	1.06 (1.02-1.10)	0.002
**Inhalation injury**	0.46 (0.17-1.28)	0.139	-	-
**CYP4F2 level**	2.93 (1.28-6.68)	0.011	3.47 (1.24-9.71)	0.018
**P2RX1 level**	2.63 (1.26-5.49)	0.010	2.16 (1.00-4.65)	0.049

TBSA: total body surface area

Table S4 MR results and sensitivity analysis

Outcome	MR results						Heterogeneity		Horizontal pleiotropy	
	Method	nSNP	b	SE	*P* value	OR (95%CI)	Q value	*P* value	Egger intercept	*P* value
CD40 ligand levels	Weighted median	18	0.127	0.128	0.321	1.135(0.884-1.459)				
	IVW	18	-0.020	0.090	0.825	0.980(0.821-1.170)				
Carcinoembryonic antigen-related cell adhesion molecule 1	Weighted median	18	-0.020	0.059	0.740	0.981(0.874-1.101)				
	IVW	18	-0.012	0.041	0.769	0.988(0.912-1.071)				
Clusterin	Weighted median	8	-0.177	0.186	0.340	0.838(0.582-1.205)				
	IVW	8	-0.348	0.135	0.010	0.706(0.541-0.920)				
DAF	Weighted median	8	0.407	0.192	0.034	1.502(1.031-2.187)			0.071	0.572
	IVW	8	0.181	0.189	0.338	1.199(0.827-1.737)	14.122	0.049		
Ectonucleoside triphosphate diphosphohydrolase 1	Weighted median	18	-0.030	0.062	0.626	0.970(0.859-1.096)				
	IVW	18	-0.017	0.041	0.680	0.983(0.907-1.065)				
Coagulation Factor VIII	Weighted median	18	0.005	0.056	0.923	1.005(0.900-1.123)				
	IVW	18	-0.002	0.041	0.953	0.998(0.921-1.081)				
Tyrosine-protein kinase Fyn	Weighted median	18	-0.025	0.058	0.674	0.976(0.870-1.094)				
	IVW	18	0.010	0.043	0.809	1.010(0.930-1.098)				
MAPK14	Weighted median	8	-0.078	0.187	0.675	0.925(0.641-1.333)				
	IVW	8	-0.111	0.180	0.539	0.895(0.629-1.275)				
Tyrosine-protein kinase Mer levels	Weighted median	19	-0.028	0.123	0.820	0.972(0.764-1.237)				
	IVW	19	-0.020	0.089	0.821	0.980(0.823-1.167)				

PIK3CA/PIK3R1	Weighted median	8	0.168	0.178	0.345	1.183(0.835-1.675)				
	IVW	8	0.043	0.135	0.750	1.044(0.802-1.359)				
Protein S	Weighted median	8	0.366	0.196	0.062	1.442(0.982-2.117)			0.056	0.54
	IVW	8	0.340	0.139	0.015	1.405(1.069-1.845)	7.392	0.389		
Tissue factor levels	Weighted median	19	-0.332	0.130	0.011	0.718(0.556-0.926)			-0.017	0.697
	IVW	19	-0.230	0.097	0.018	0.794(0.657-0.961)	21.192	0.27		
TFPI	Weighted median	8	0.180	0.171	0.293	1.197(0.856-1.674)			0.129	0.150
	IVW	8	0.272	0.136	0.045	1.313(1.007-1.713)	7.427	0.386		

MR: Mendelian randomization; IVW: Inverse Variance Weighted; DAF: decay accelerating factor; TFPI: tissue factor pathway inhibitor

Table S5 Comparison of Patients' demographics and baseline characteristics among different cohorts

Characteristic	transcriptomic cohort		Retrospective cohort	*P* value[2]
	GSE77791, n = 15[1]	GSE19743, n = 57[1]	n = 583[1]	
**Sex-male**	9 (60%)	45 (79%)	409 (70%)	0.242
**Age(year)**				0.400
**<40**	6 (40%)	24 (42%)	176 (30%)	
**40-59**	6 (40%)	24 (42%)	289 (50%)	
**≥60**	3 (20%)	9 (16%)	118 (20%)	
**TBSA (%)**	70 (44, 76)	66 (52, 79)	60 (45, 80)	0.081
**Inhalation injury**	-	37 (65%)	433 (74%)	0.156
**Death**	2 (13%)	15 (26%)	86 (15%)	0.077
**Location**	France	USA	China	

[1]n (%); Median (IQR); [2]Kruskal-Wallis rank sum test; Fisher's exact test.

TBSA: total body surface area

Table S6: Variables screened by three algorithms

Variables	Univariable		LASSO		Stepwise-backward		Elastic Net	
	**HR (95%CI)**	***P* value**	**HR (95%CI)**	***P* value**	**HR (95%CI)**	***P* value**	**HR (95%CI)**	***P* value**
Age	1.05 (1.03-1.00)	<0.001	-	-	1.05 (1.03-1.06)	<0.001	-	-
TBSA	1.04 (1.03-1.00)	<0.001	1.03 (1.02-1.05)	<0.001	1.04 (1.02-1.05)	<0.001	1.03 (1.02-1.05)	<0.001
Inhalation injury(Yes)	1.57 (1.02-2.50)	0.038	-	-	-	-	-	-
Prothrombin time	1.05 (1.03-1.00)	<0.001	1.07 (1.00-1.14)	0.069	-	-	1.07 (1.00-1.14)	0.063
APTT	1.03 (1.01-1.00)	<0.001	-	-	-	-	-	-
Thrombin time	1.04 (1.00-1.00)	0.064	0.93 (0.87-0.99)	0.029	-	-	0.93 (0.87-0.99)	0.028
D-dimer	1.02 (1.01-1.00)	<0.001	1.02 (1.01-1.03)	**0.001**	1.01 (1.00-1.02)	**0.038**	1.02 (1.01-1.03)	**0.001**
INR	4.09 (1.93-8.00)	<0.001	0.57 (0.16-1.96)	0.372			0.57 (0.16-1.96)	0.352
Fibrinogen	0.94 (0.79-1.90)	0.42	-	-	-	-	-	-
Calcium	1.57 (0.88-2.50)	0.125	2.98 (1.43-6.23)	**0.004**	3.83 (1.79-8.19)	**0.001**	2.98 (1.43-6.23)	**0.004**

PH	0.01 (0.00-0.00)	<0.001	0.35 (0.03-4.37)	0.414	-	-	0.35 (0.03-4.37)	0.382
Lactic acid	1.11 (1.05-1.10)	<0.001	1.04 (0.98-1.11)	0.165	1.08 (1.01-1.14)	0.021	1.04 (0.98-1.11)	0.167
Total bilirubin	1.01 (1.00-1.00)	0.027	1.01 (1.00-1.03)	0.081	1.01 (1.00-1.03)	0.067	1.01 (1.00-1.03)	0.07
Creatinine	1.01 (1.01-1.00)	<0.001	1.01 (1.01-1.02)	<0.001	1.01 (1.01-1.01)	<0.001	1.01 (1.01-1.02)	<0.001
Albumin	0.96 (0.94-0.90)	0.003	0.98 (0.95-1.02)	0.354	0.97 (0.94-1.00)	0.066	0.98 (0.95-1.02)	0.333
WBC	1.03 (1.01-1.00)	0.003	-	-	-	-	-	-
Platelet	1.00 (1.00-1.00)	0.003	1.00 (1.00-1.00)	0.326	-	-	1.00 (1.00-1.00)	0.286

TBSA: total body surface area; APTT: activated partial thromboplastin time; INR: international normalized ratio; WBC: white blood cell

Table S7 Mediation analysis of D-dimer and calcium for the associations between TBSA and death

Mediator	Total effect		Indirect effect		Direct effect		Proportion mediated, % (95% CI)
	Coefficient (95% CI)	*P* value	Coefficient (95% CI)	*P* value	Coefficient (95% CI)	*P* value	
D-dimer	0.00058 (0.00026, 0.00090)	<0.001	0.00002 (0.00000, 0.00007)	0.048	0.00057 (0.00025, 0.00087)	<0.001	2.9 (0.0, 9.4)
Calcium	0.00060 (0.00028, 0.00092)	<0.001	0.00001 (-0.00003, 0.00004)	0.56	0.00060 (0.00027, 0.00090)	<0.001	1.1 (-4.1, 6.1)

Indirect effect: mediation effect of D-dimer or calcium between TBSA and death. Direct effect; association between TBSA and death without mediation. The mediation analyses were adjusted for age, TBSA, inhalation injury, PH, lactic acid, total bilirubin, creatinine, albumin and WBC

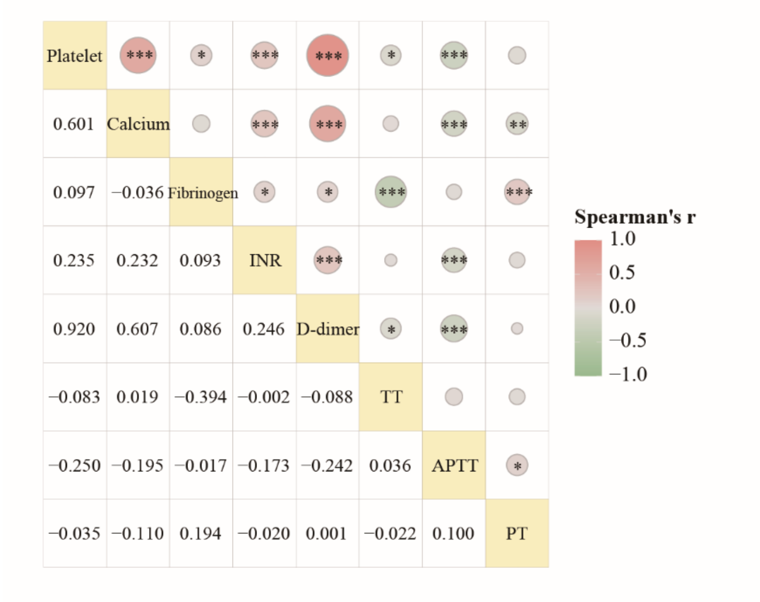

Figure S2 Correlation between coagulation related variables. *:*P*<0.05; **: *P*<0.01; ***: *P*<0.001. INR: international normalized ratio; TT: Thrombin time; APTT: activated partial thromboplastin time; PT: Prothrombin time

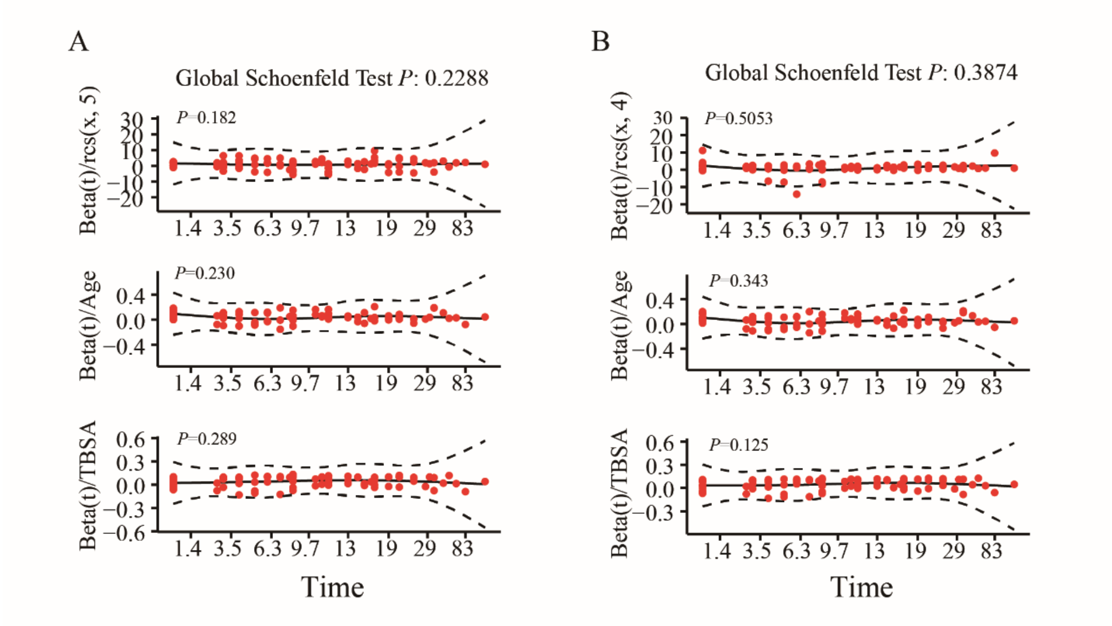

Figure S3 Schoenfeld test for Cox proportional-hazards model. *P* value >0.05 indicated that the survival risk does not change over time. The Cox model is considered to be consistent with the proportional risk assumption.
